# Ubiquitin Conjugating Enzyme E2 H (UBE2H) Is Linked to Poor Outcomes and Metastasis in Lung Adenocarcinoma

**DOI:** 10.3390/biology10050378

**Published:** 2021-04-28

**Authors:** Meng-Chi Yen, Kuan-Li Wu, Yu-Wei Liu, Yung-Yun Chang, Chao-Yuan Chang, Jen-Yu Hung, Ying-Ming Tsai, Ya-Ling Hsu

**Affiliations:** 1 Department of Emergency Medicine, Kaohsiung Medical University Hospital, Kaohsiung Medical University, Kaohsiung 807, Taiwan; yohoco@gmail.com; 2 Graduate Institute of Medicine, College of Medicine, Kaohsiung Medical University, Kaohsiung 807, Taiwan; 980448kmuh@gmail.com (K.-L.W.); chaoyuah@kmu.edu.tw (C.-Y.C.); yainghsu@kmu.edu.tw (Y.-L.H.); 3 Division of Pulmonary and Critical Care Medicine, Kaohsiung Medical University Hospital, Kaohsiung Medical University, Kaohsiung 807, Taiwan; jyhung@kmu.edu.tw; 4 Drug Development and Value Creation Research Center, Kaohsiung Medical University, Kaohsiung 807, Taiwan; 5 Division of Thoracic Surgery, Department of Surgery, Kaohsiung Medical University Hospital, Kaohsiung Medical University, Kaohsiung 807, Taiwan; Nipma6714@gmail.com; 6 Division of Internal Medicine, Kaohsiung Medical University Hospital, Kaohsiung Medical University, Kaohsiung 807, Taiwan; cyy807@gmail.com; 7 Department of Anatomy, Kaohsiung Medical University, Kaohsiung 807, Taiwan; 8 Department of Internal Medicine, Kaohsiung Municipal Ta-Tung Hospital, Kaohsiung Medical University, Kaohsiung 807, Taiwan; 9 Department of Respiratory Care, College of Medicine, Kaohsiung Medical University, Kaohsiung 807, Taiwan; 10 Department of Internal Medicine, School of Medicine, College of Medicine, Kaohsiung Medical University, Kaohsiung 807, Taiwan

**Keywords:** lung adenocarcinoma, metastasis, hypoxia, ubiquitin conjugating enzyme E2 H (UBE2H), RNA sequencing, microRNA

## Abstract

**Simple Summary:**

The development of novel treatments for metastatic lung adenocarcinoma is an important issue because some patients do not respond to current standard therapies. Our study aimed to investigate the gene expression profiles in non-tumor tissue, primary tumor tissue, and the metastatic lung tumor tissue in the pleura. After RNA sequencing and bioinformatic analysis from a patient with lung adenocarcinoma (LUAD), ubiquitin conjugating enzyme E2 H (UBE2H) was identified. Compared with normal tissue, a higher expression of UBE2H was observed in the tumor tissue. The high UBE2H expression was significantly associated with poor survival. Suppressing UBE2H in cell lines of lung adenocarcinoma inhibited metastatic capacity and reversed epithelial–mesenchymal transition signaling pathway. Five microRNAs, including miR-101, miR-30a, miR-30b, miR-328, and miR-497, predicted to target UBE2H might be potential prognostic biomarkers for survival in lung adenocarcinoma. The copy number variation may be involved in the regulation of the UBE2H expression. Our observations show that UBE2H is a novel regulatory molecule of metastasis, and may be a prognostic biomarker and a therapeutic target in lung adenocarcinoma.

**Abstract:**

The prognosis of patients with metastatic lung adenocarcinoma (LUAD) is poor. Although novel lung cancer treatments have been developed for metastatic LUAD, not all patients are fit to receive these treatments. The present study aimed to identify the novel regulatory genes in metastatic LUAD. Because the pleural cavity is a frequent metastasis site of LUAD, the adjacent non-tumor tissue, primary tumor tissue, and metastatic lung tumor tissue in the pleura of a single patient with LUAD were collected. The gene expression profiles of the collected samples were further analyzed via RNA sequencing and bioinformatic analysis. A high expression level of ubiquitin conjugating enzyme E2 H (UBE2H), a hypoxia-mediated gene, was identified in the metastatic malignant pleural tumor. After accessing the survival data in patients with lung adenocarcinoma through online databases, a high UBE2H expression was associated with poor survival for LUAD. UBE2H knockdown in two lung adenocarcinoma cell lines suppressed the cell migration capacity and reversed the epithelial–mesenchymal transition (EMT) signaling pathway. A high expression of UBE2H-targeting microRNAs, including miR-101, miR-30a, miR-30b, miR-328, and miR-497, were associated with a favorable prognosis. Moreover, the UBE2H expression revealed a significant correlation with the copy number variation. Taken together, the presence of UBE2H regulated the EMT program and metastasis in LUAD.

## 1. Introduction

Lung cancer is one of the leading cancer types for the estimated new cancer cases and deaths according to the cancer statistics [1]. Non-small cell lung cancer (NSCLC; approximately 85% of patients) and small cell lung cancer (SCLC; approximately 15% of patients) are the two main forms of lung cancer, and NSCLC is classified into three types, namely adenocarcinoma, squamous cell carcinoma, and large cell lung cancer [2]. A World Health Organization report reveals adenocarcinoma is the most common type of NSCLC [3]. When patients are diagnosed with metastatic NSCLC, the five-year overall survival rate is less than 5% [4]. Immune checkpoint inhibitors (ICIs) and targeted therapies have emerged in the last decade. The specific genetic alterations, including *epidermal growth factor receptor* (*EGFR*) gene mutation (30–40% of Asians and 10–20% of Caucasians), *echinoderm microtubule associated protein like* 4(*EML4*)-*anaplastic lymphoma kinase* (*ALK*) gene fusions (up to 7%), ROS proto-oncogene receptor tyrosine kinase 1 (ROS1) gene rearrangements (1.7%), BRAF V600E gene mutation (2%), and Programmed death-ligand 1 (PD-L1) status, are examined in patients with metastatic diseases in order to choose an adequate ICI [5,6]. These treatments improve the clinical outcomes in some patients with metastatic NSCLC [6]. However, some patients are not able to receive these treatments of they do not have genetic alterations in above targeted genes [6]. Therefore, investigating the molecular mechanisms of metastatic lung cancer is still a crucial issue.

Besides the molecular alterations of NSCLC, the metastasis of NSCLC is driven by various factors. For example, interactions between cancer cells and fibroblasts, stromal cells, and endothelial cells remodels the extracellular matrix [7]. The tumor-infiltrating immune cells secret soluble factors and regulate the immune environment [8]. Hypoxic and metabolic stress also alter the metastatic cascade in malignant cells [9,10]. These factors are thought to trigger the signaling pathways of epithelial–mesenchymal transition (EMT) and to prime the premetastatic niche [11]. There are some limitations in the current targeted therapies and immunotherapies. To develop novel treatments for metastatic NSCLC, investigation of the detailed mechanisms in metastatic NSCLC is essential.

Cancer is one of the causes of pleural effusion [12]. Some soluble molecules in pleural effusion are used as diagnostic markers to identify whether the fluid is malignant pleural effusion [13,14]. The pleural cavity is a frequent metastatic site of lung cancer [15]. Malignant pleural effusion is observed in approximately 50% of patients with advanced NSCLC [16]. The molecular profiling of the mutation status in NSCLC and malignant pleural effusions with next generation sequencing (NGS) provides useful information for cancer management [17,18]. Furthermore, transcriptome analysis in tumor samples via RNA sequencing can identify novel biomarkers and predict signaling pathways. The present study sought to identify novel regulatory genes in metastatic lung adenocarcinoma by utilizing the tissues of the adjacent non-tumor tissue, primary tumor tissue, and metastatic lung tumor tissue in the pleura from a patient. The collected samples were further analyzed for their gene expression profiles via RNA sequencing, bioinformatic analysis, and experimental validation.

## 2. Materials and Methods

### 2.1. Sample Collection

The present study was approved by the Institutional Review Board of Kaohsiung Medical University Hospital (IRB no. KMUHIRB-G(II)-20180021). The samples were collected from non-tumor tissue and the tumor tissue of the primary tumor, as well as tumor cells in pleural effusion from an Asian male patient in his 50s with lung adenocarcinoma, staged as T4N0M1a.

### 2.2. RNA Sequencing

The RNA of each sample was extracted using TRIzol^®^ reagent (Thermo Fisher Scientific, Inc., Madison, WI, USA). The RNA quality, RNA sequencing, small RNA sequencing, sequencing quality trimming, and mRNA alignment were accomplished by Welgene Biotech Co., Ltd. (Welgene Biotech, Taipei, Taiwan). The p-value of the differential genes was calculated using cuffdiff with a non-grouped sample using “blind mode” [19].

### 2.3. Bioinformatic Analysis

The genes with a differential expression (fold change > 2; *p* value < 0.05) were subjected to a gene set enrichment analysis (http://www.broad.mit.edu/gsea/; GSEA version 6.2; 1 October 2018) [20,21]. The UBE2H expression in the different stages of the lung adenocarcinoma (LUAD) samples of the Cancer Genome Atlas (TCGA) was analyzed from UALCAN website (http://ualcan.path.uab.edu; 1 November 2020) [22]. The correlation of UBE2H with various proteins was assessed using the Clinical Proteomic Tumor Analysis Consortium (CPTAC) database (https://proteomics.cancer.gov/programs/cptac; 1 November 2020). The association between the UBE2H expression and survival rate, as well as the microRNA expression and survival rate, was analyzed via the Kaplan–Meier plotter (KM plotter) (http://kmplot.com/; 1 October 2018) [23] and SurvExpress (http://bioinformatica.mty.itesm.mx:8080/Biomatec/SurvivaX.jsp; 1 October 2018) [24]. UBE2H targeted microRNAs were predicted through the miRWalk2.0 website (http://zmf.umm.uni-heidelberg.de/apps/zmf/mirwalk2/; 1 October 2018) [25]. The association between the UEB2H expression and each microRNA expression was evaluated via the dataset of TCGA-LUAD.

### 2.4. Cell Culture and Transfection

Human cell line A549 (ATCC^®^ CCL-185™, Manassas, Virginia, USA) and EML4-ALK Fusion-A549 Isogenic Cell Line Human (ATCC^®^ CCL-185IG™, Manassas, VA, USA) were obtained from the American Type Culture Collection (ATCC; Manassas, VA, USA). Both cells were maintained in an F-12K medium supplied with 10% fetal bovine serum (FBS) and 1% penicillin-streptomycin (Thermo Fisher Scientific, Madison, WI, USA) in a 37 °C, 5% CO_2_ incubator. The cells were transfected with of 1 μg UBE2H shRNA (clone ID, TRCN0000314525, targeting sequence: 5′-TACGATCCTGGGAGGACTTAA-3′) or control shRNA (non-specific shRNA) and were obtained from the National RNAi Core Facility (Taipei, Taiwan) via lipofectamine 2000 (Invitrogen), according to the manufacturer’s instructions.

### 2.5. qPCR

The RNA extraction was done using TRIzol reagent (Thermo Fisher Scientific, Waltham, MA, USA). The RNA reverse transcription was performed with a PrimeScript RT reagent kit (Clontech Laboratories, Inc., Mountainview, CA, USA). The PCR product was detected using the specific primers of UBE2H forward, 5′-ATGTACCTCCACCGACCAGA-3′ and reverse, 5′-ACTCCATATCCTGGGCCTCA-3′; GAPDH forward, 5′-GAGTCAACGGATTTGGTCGT-3′ and reverse; and 5′-TTGATTTTGGAGGGATCTCG-3′ on a StepOne Plus Real-Time PCR System (Applied Biosystems; Thermo Fisher Scientific, Inc., Austin, TX, USA) using the Fast SYBR Green Master Mix (Applied Biosystems; Thermo Fisher Scientific, Inc., Austin, TX, USA)). The relative mRNA expression levels were calculated using the 2 ^−ΔΔCt^ method [26] and GAPDH served as an internal control.

### 2.6. Western Blot

The protein lysates of A549 and EML4-ALK fusion-A549 Isogenic cell were collected in a radioimmunoprecipitation lysis (RIPA) buffer with a protease inhibitor (Millipore) and the protein concentration was determined with a bicinchoninic acid (BCA) protein assay kit (Thermo Fisher Scientific, Rockford, IL, USA). Each protein was incubated with a primary antibody, including anti-GAPDH (1:5000, cat. no. MAB374; Millipore), anti- N-cadherin (1:1000, cat. no. 610920), anti-E-cadherin (1:1000, cat. no. 610182), anti-Vimentin (1:1000, cat. no.550513) BD Biosciences, San Jose, CA, USA), anti-Snail (1:1000, cat. no. #3879, Cell Signaling Technology, Danvers, MA, USA), and anti-UBE2H (1:500, cat. no. E2-607, BostonBiochem) at 4 °C overnight. The blots were visualized with the Alpha Innotech FluorChem FC2 imaging system (ProteinSimple; Bio-Techne, Minneapolis, MN, USA).

### 2.7. Wound-Healing Assay

The cells were seeded into a 24 well plate and a scratch was made by a 200 μL pipette tip when the cells reached a 90–100% confluent monolayer. After removing the cell debris through phosphate-buffered saline washing, the cells were incubated in a culture medium with 2% FBS for 24 h.

### 2.8. Transwell Migration Assay

The cells were seeded into a QCM™ 24-well Cell Migration Assay and Invasion System (Merck KGaA, Darmstadt, Germany) with uncoated 8 μm pore size polycarbonate membranes (Millipore) in 300 μL of a serum free medium. Then, 500 μL of a medium with 10% FBS was placed in the lower chamber of a 24 well plate. After 24 h, the bottom of the membrane was fixed in 4% formaldehyde and then stained in 1% crystal violet. A cotton swab was used to remove the cells on the upper surface. The stained cells in the bottom of the membrane were then visualized using the Olympus inverted microscopes. Four random fields of view were counted and the relative fold of migration in each group was compared with the control group.

### 2.9. Statistics

The bar graphs and statistics were performed by GraphPad Prism 7 (GraphPad Software, San Diego, CA, USA). An unpaired, two-tailed Student’s t test was used for analyzing the difference between the two groups. A *p*-value < 0.05 was considered to indicate a statistically significant difference.

## 3. Results

### 3.1. Identification of Ubiquitin Conjugating Enzyme E2 H (UBE2H) in the Malignant Pleural of Lung Adenocarcinoma

**Figure 1:**
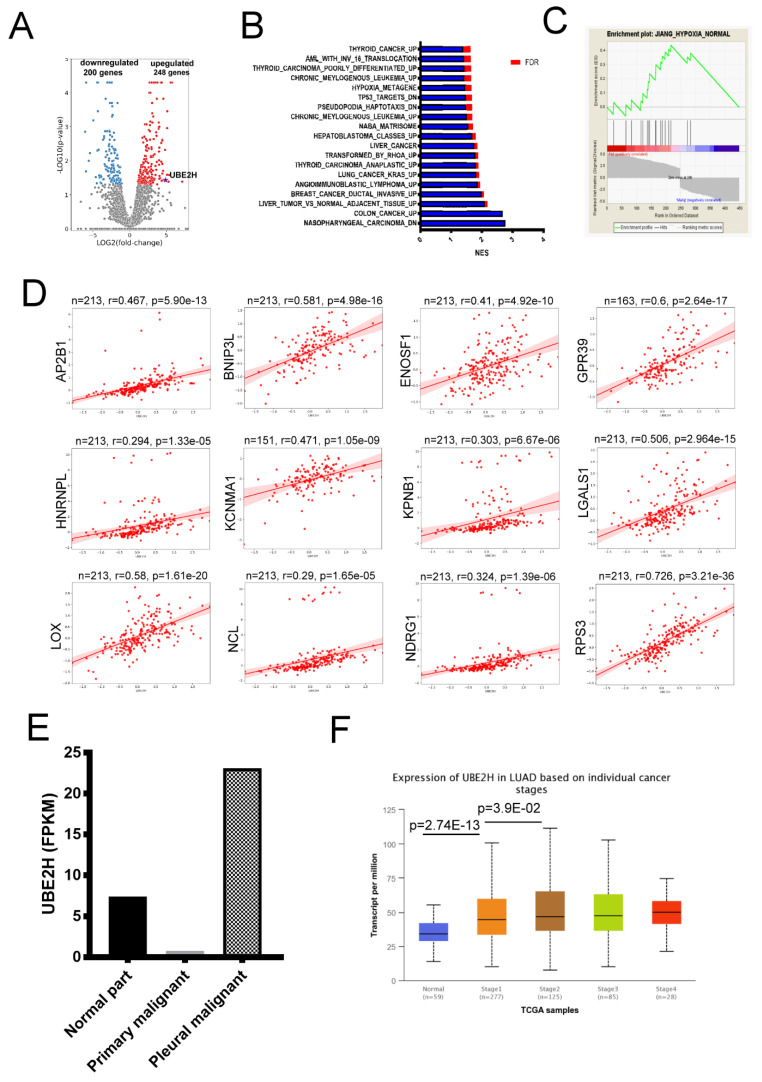
Identification of ubiquitin conjugating enzyme E2 H (UBE2H) from lung adenocarcinoma (LUAD) tumor samples. (**A**) The volcano plot (log2 fold-change vs. log10 *p*-value) of upregulated and downregulated genes showed the differential expression distribution for the sample pair. The green and red dots indicate significant down- and up-regulated genes, respectively. (**B**) The enriched biological processes by gene set enrichment analysis. (**C**) The histogram plot of hypoxia-related gene set. (**D**) The correlation of UBE2H with the genes of the hypoxic pathway. (**E**) The expression of UBE2H in the samples collected in the present study. (**F**) The expression of UBE2H in the lung adenocarcinoma (LUAD) samples of the Cancer Genome Atlas (TCGA). NES-normalized enrichment score; FDR-false discovery rate; FPKM-fragments per kilobase of transcript per million.

The samples were collected from a patient with LUAD. The RNA of the adjacent non-tumor tissue, primary tumor tissue, and metastatic lung tumor tissue in the pleura were performed by RNA sequencing. The genes with a differential expression are shown in Figure 1A. The full gene list is shown in Supplementary Table S1. After the gene set enrichment analysis, the enriched pathways were observed (Figure 1B,C). Hypoxic stress is an important factor to induce the metastasis of cancer [9], and the list and fold change of the genes involved in the hypoxia-related gene set are shown in Table 1. Ubiquitin conjugating enzyme E2 H (UBE2H) is observed in Table 1. Currently, the role of UBE2H is almost unknown in hypoxic-related pathways. However, the correlation analysis showed a high positive association of UBE2H with genes of a hypoxic pathway (AP2B1, BNIP3L, ENOSF1, GPR31, HNRNPC, KCNMA1, KPNB1, LGALS1, LOX, NCL, NDRG1, and RPS3; Figure 1D). The present study focused on the role of UBE2H in LUAD. According to the expression data (fragments per kilo base per million mapped reads (FPKM)) from the RNA sequencing, the highest expression and the lowest expression of UBE2H were observed in the malignant pleural and the primary tumor tissue, respectively (Figure 1E). The UBE2H expression in other patients with LUAD in different tumor stages was evaluated in the LUAD samples of the Cancer Genome Atlas (TCGA). In Figure 1F, a significantly higher UBE2H expression in stages 1 to 4 was observed when compared with the normal tissues (normal vs. stage 1: *p*-value = 2.7 × 10^−13^; normal vs. stage 2: *p*-value = 2.4 × 10^−11^; normal vs. stage 3: *p*-value = 2.6 × 10^−10^; and normal vs. stage 4: *p*-value = 1.6 × 10^−4^). In addition, a statistical significance between stage 1 vs. stage 2 was observed (*p*-value = 3.9 × 10^−2^), but no statistical significance was observed between the other tumor stages. The results indicated that the UBE2H expression was higher in all LUAD samples when compared with the normal samples.

### 3.2. Investigation of the Association between UBE2H Expression and Survival in Patients with LUAD

**Figure 2:**
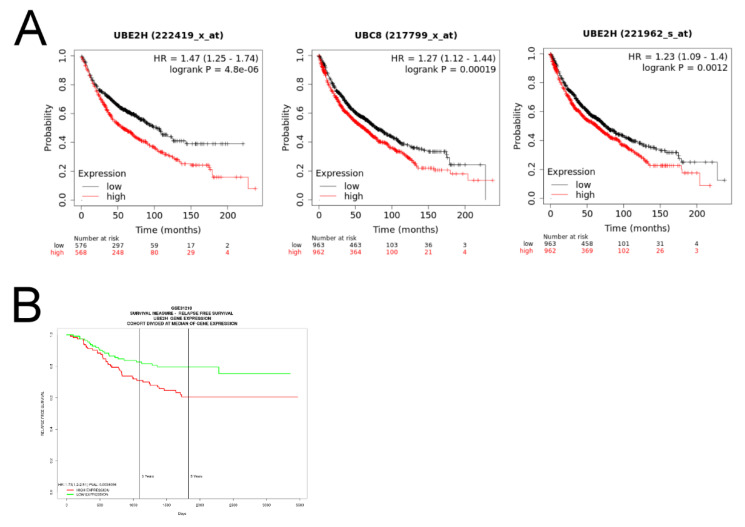
The correlation of UBE2H expression and survival in patients with LUAD. (**A**) The survival analysis via the KM plotter. The samples are divided into high and low expressions according to median expression value of UBE2H. The red and black lines indicate high and low UBE2H expressions, respectively. Three plots are analyzed by the different probes labeled on each plot. (**B**) The survival analysis via GSE31210 dataset on SurvExpress. The red and green lines indicate high and low UBE2H expressions, respectively. HR—hazard ratios.

As high expression levels of UBE2H were observed in the tumor samples of LUAD, we further investigated whether the expression levels of UBE2H were associated with the survival of patients through the online database of the KM plotter [23]. The results are shown in Figure 2A. UBE2H detected by three different probes was significantly associated with a poor survival. In another database (the data adapted from GSE31210) [24,27], a similar trend was observed (Figure 2B). This evidence supports that UBE2H may be associated with a poor prognosis in LUAD.

### 3.3. Investigating the Role of UBE2H in Migration Capacity of LUAD Cell Lines

**Figure 3:**
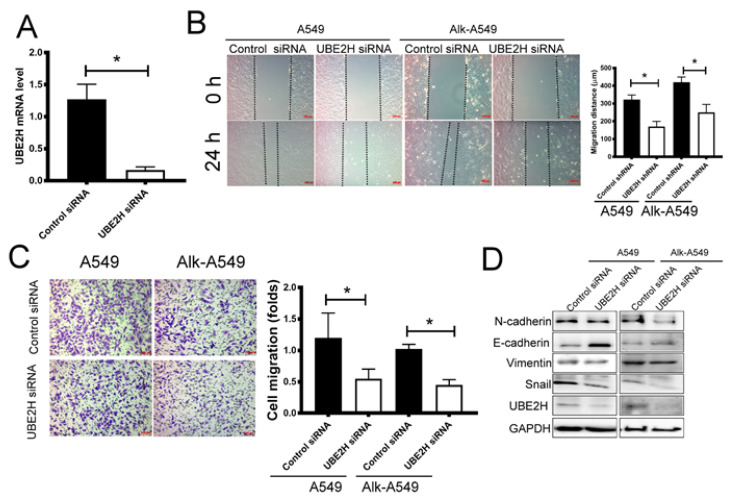
The effect of UBE2H silencing on the cell migratory capacity in LUAD cell lines. (**A**) The effect of UBE2H shRNA is examined in A549. (**B**) Wound-healing assay (*n* = 3). (**C**) Transwell migration assay and quantitative results (*n* = 3). (**D**) Western blot assay for determining the regulatory molecules of the epithelial–mesenchymal transition signaling pathway (*n* = 3). * *p*-value < 0.05, as compared with the vector control.

To determine the role of UBE2H in LUAD, two LUAD cell lines, A549 and EML4-ALK fusion-A549, were used. The *EML4-ALK* fusion gene, which is identified in approximately 4–6% of LUAD patients, enhances neoplastic phenotypes because the mutation causes continuous ALK activation [28]. EML4-ALK-A549 is derived from the A549 parental cell line containing *EML4-ALK* fusion variant 1. The effect of UBE2H shRNA is shown in Figure 3A. The wound healing assay and transwell migration assay revealed that the migration capacity of A549 and EML4-ALK-A549 was suppressed after the transfection of UBE2H shRNA (Figure 3B,C). The epithelial–mesenchymal transition (EMT) is an important signaling pathway for regulating cell migration [29]. The key regulatory molecules were detected by Western blot. The mesenchymal phenotype-associated molecules including N-cadherin and Snail in UBE2H knockdown A549 and EML4-ALK-A549 cells was lower than that in the control cells (Figure 3D). By contrast, the expression of Vimentin was not significantly changed. The results suggest that UBE2H knockdown can suppress the metastatic capacity in LUAD through the downregulation of Snail, N-cadherin, and E-cadherin.

### 3.4. The Potential Regulatory Mechanisms for UBE2H Expression in LUAD

**Figure 4:**
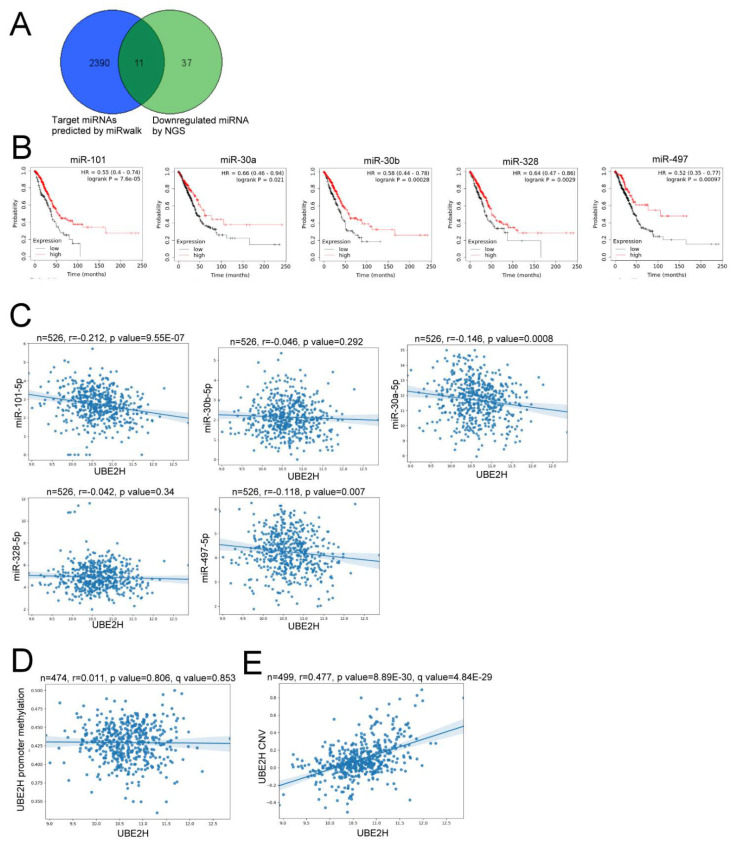
The potential regulatory mechanism of UBE2H. (**A**) 11 shared genes are identified from the 2390 candidate UBE2H-targeted miRNAs (blue circle) predicted by miRWalk and 37 down-regulated miRNAs from RNA sequencing (green circle). (**B**) The survival analysis via the KM plotter. The samples are divided by the median expression value of each miRNA. (**C**) The correlation between the expression of UBE2H and the expression of each miRNA. (**D**) The correlation between the expression of UBE2H and the methylation status of the UBE2H promoter. (**E**) The correlation between the expression of UBE2H and the copy number variation (CNV) among the LUAD samples of TCGA.

As the upregulation of UBE2H was associated with a poor prognosis and metastatic function in LUAD, the mechanism of UBE2H gene expression was further investigated. Because gene expression can be regulated by microRNA (miRNAs), the potential UBE2H-targeted miRNAs were predicted (Figure 4A). Here, 2390 UBE2H-targeted miRNAs were predicted by the miRWalk database [25]; besides, the results of RNA sequencing in the present study identified 37 downregulated miRNAs (Figure 4A). Of these, 11 shared miRNAs were observed, and the list and fold change are shown in Table 2. Among the 11 miRNAs, a relatively high expression of miR-101, miR-30a, miR-30b, miR-328, and miR-497 was significantly associated with good survival in patients with LUAD through the KM plotter database (Figure 4B). However, only a very weak correlation was observed, even though the *p*-value reached a statistically significant value after evaluating the expression profiles in the LUAD samples of TCGA (Figure 4C). Furthermore, the UBE2H expression was not correlated with the methylation status of the UBE2H promoter (Figure 4D), but was significantly correlated with the copy number variation (CNV; Figure 4E). These results might suggest that CNV, but not miRNAs or methylation status, is an important factor to up-regulate UBE2H expression.

## 4. Discussion

Lung cancer has had a notoriously high fatality rate for decades. The novel strategy, RNA sequencing, provides an advanced technique to discover the genes of interest. In this study, we utilized a lung cancer patient with pleural metastasis to discover a gene of interest, UBE2H. Through a public database, a higher expression of UBE2H conferred a poor survival outcome. The progression and invasion of lung cancer were attenuated via the knockdown of UBE2H. Furthermore, the regulatory mechanisms in the UBE2H expression were considered via epigenetic regulation. The findings suggest that UBE2H plays a role in tumor metastasis and can be of therapeutic application in lung cancer.

RNA sequencing was used to identify the novel regulatory genes for the metastatic phenotypes of LUAD. Hundreds of genes with a differential gene expression between primary tumor and malignant pleural were identified, and these genes are involved in multiple biological pathways. The hypoxia-related pathway was focused on it is an important factor affecting the behavior of tumor cells in the tumor microenvironment [9,10]. Among the 14 genes of the hypoxia-related gene set (Table 1), the UBE2H gene was chosen. UBE2H belongs to the ubiquitin conjugating enzyme (UBE2) family, which are involved in ubiquitination and proteasome-mediated protein degradation [30]. There are 40 members in the UBE2 family. Emerging evidence reveals that the aberrant expression of some UBE2 family members is observed in tumor samples. A high expression of UBE2S is associated with malignant breast cancer [31], and an overexpression of UBE2S promotes the proliferation and survival in LUAD cells [32]. The transfection of the UBE2C gene enhances cell proliferation and invasion in lung cancer cells [33], and the expression of UBE2C serves as a prognostic gene for lung adenocarcinoma [34]. The roles of UBE2H in human disease are not well-known. The association of UBE2H and sporadic amyotrophic lateral sclerosis has been reported [35]. Circulating UBE2H mRNA is a potential diagnostic marker or therapeutic target of treatment for Alzheimer’s disease [36]. UBE2H is one of the ubiquitination-related genes used to predict the prognosis of pancreatic cancer patients [37]. In addition, the overexpression of UBE2H enhances proliferation in murine hepatoma cells [38]. Currently, to the best of our knowledge, only one case report indicates that the MET-UBE2H gene fusion is associated with epidermal growth factor receptor (EGFR) resistance in LUAD [39]. Therefore, UBE2H was focused on in the present study.

The RNA sequencing data showed a relatively high UBE2H expression in the metastatic lung tumor tissue in the pleura (Figure 1D). In Figure 1E, the UBE2H expressions in stages 1 to 4 of the LUAD samples of TCGA are higher than in the normal samples. A high expression of UBE2H was associated with poor survival in patients with LUAD (Figure 2). Moreover, the UBE2H expression in stage 2 was significantly higher than that in stage 1 lung cancer. Although the expression of UBE2H did not reveal a statistical difference between stages 3 or 4 vs. stage 1, the mean UBE2H expression in stages 3 and 4 was higher compared with stage 1 (Figure 1E). This might imply that the upregulation of UBE2H is associated with tumor metastasis, even though it is in the early stage of metastasis. UBE2H has been observed in 75-gene signatures associated with lymph node metastasis in lung adenocarcinoma [40]. UBE2H has been identified in metastasis-associated gene sets in colorectal cancer [41]. Our results demonstrated that suppressing the UBE2H expression of A549 and EML4-ALK-A549 reduced the metastatic capacity. The regulatory molecules of the EMT pathways, including Snail, N-cadherin, and E-cadherin, were affected after silencing UBE2H (Figure 3). The ubiquitin–proteasome signaling pathway is an important factor for regulating the EMT phenotype [42], but how UBE2H affects EMT signaling is still unknown. This detailed mechanism should be investigated in the future.

miRNAs are involved in the regulation of the gene expression and various biological processes including metastasis [43]. According to the RNA sequencing in the present study and candidate UBE2H targeting miRNAs, we observed that the expression miR-101, miR-30a, miR-30b, miR-328, and miR-497 was associated with a good survival. mir-101, mir-30a, and miR-30b have been demonstrated to be suppressor miRNAs for lymph node metastasis in NSCLC [44,45]. However, no significant correlation between the UBE2H expression and miRNAs were observed in the LUAD samples of TCGA (Figure 4C,D). Our results implied that the copy number variation is a potential factor to increase UBE2H expression in TCGA samples (Figure 4E). Currently, no further experimental evidence or studies provide a regulatory mechanism between the copy number variation and the expression of UBE2H. This should be a critical issue to be investigated in the future.

## 5. Conclusions

**Figure 5:**
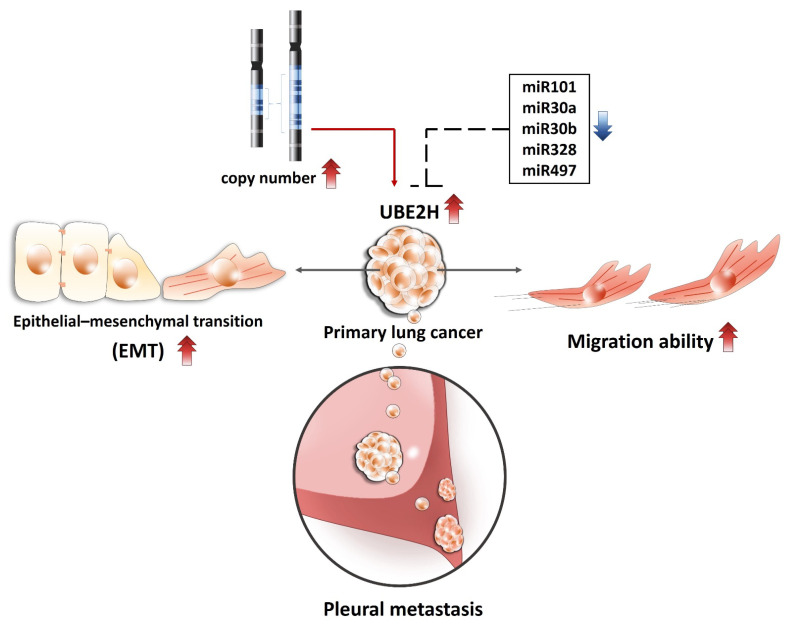
The proposed mechanisms for how UBE2H regulates lung cancer EMT and metastasis and UBE2H is regulated.

Collectively, the present study identified a novel hypoxia-related gene UBE2H from the malignant pleural of a patient with LUAD, investigated the role of UBE2H in metastatic capacity in two LUAD cell lines, and predicted a potential mechanism for the regulation of the UBE2H expression (Figure 5). The current findings demonstrate that UBE2H serves as a potential prognostic biomarker and a therapeutic target for metastatic LUAD.

## Figures and Tables

**Table 1:** The expression of the specific genes in the hypoxia-related gene set.

Gene Symbol	Log2 (Fold) (Pleural Malignancy/Primary Malignancy)	Gene Symbol	Log2 (Fold) (Pleural Malignancy/Primary Malignancy)
LGALS1	18.337	UBE2H	4.889
AP2B1	4.034	ENOSF1	3.074
NCL	2.996	KPNB1	2.816
RPS3	2.768	KCNMA1	2.708
LOX	2.304	HNRNPC	2.286
HIF1A	2.114	BNIP3L	2.087
NDRG1	1.963	GPR39	1.770

**Table 2:** The candidate miRNAs regulating UBE2H.

miRNA	Fold (Pleural Malignant/Primary Malignant)	miRNA	Fold (Pleural Malignant/Primary Malignant)
hsa-miR-101-3p	0.442752	hsa-miR-122-5p	0.000752
hsa-miR-1275	0.486842	hsa-miR-135a-5p	0.035668
hsa-miR-143-3p	0.323242	hsa-miR-30a-3p	0.167051
hsa-miR-30b-3p	0.410777	hsa-miR-3150b-5p	0.000195
hsa-miR-497-3p	0.142195	hsa-miR-328-3p	0.477685
hsa-miR-6724-5p	0.413534		

## Supplementary Materials

The following are available online at https://www.mdpi.com/article/10.3390/biology10050378/s1, Table S1. The full list of genes with significant changes.
